# Defects in Graphene/h-BN Planar Heterostructures: Insights into the Interfacial Thermal Transport Properties

**DOI:** 10.3390/nano11020500

**Published:** 2021-02-16

**Authors:** Wenjuan Yao, Lei Fan

**Affiliations:** School of Mechanics and Engineering Science, Shanghai University, Shanghai 200072, China; fanleigl@shu.edu.cn

**Keywords:** fraphene/h-BN planar heterostructures, multi-field coupling, defects, interfacial configuration, interfacial thermal transport

## Abstract

In this work, the defects (local stress generated) induce the formation of graphene/h-BN planar heterostructure (Gr-hBN-PH) to form "unsteady structure". Then, the coupling effects of external field (heat flow direction, strain and temperature field) and internal field (defect number, geometry shape and interfacial configuration) on the interface thermal conductivity (ITC) of Gr-hBN-PH were studied. The results show phonon transmission is less affected by compression deformation under the action of force-heat-defect coupling, while phonon transmission of heterostructure is more affected by tensile deformation. The non-harmonic interaction of the atoms in the composite system is strengthened, causing the softening of high-frequency phonons. The greater reduction of thermal transport at the interface of heterostructures will be. The interface bonding morphology plays a significant role on the ITC of the Gr-hBN-PH. The relationship between structure and properties in the low dimension is analyzed from the perspective of defect energy. It is helpful for us to understand the physical mechanism of low-dimensional structure, realize multiple structural forms, and even explore new uses.

## 1. Introduction

The in-plane hybrid structure involves the seamless splicing of two or more different atomic monomolecular films together through covalent bonds [1,2,3]. The special connection mode may create many interesting thermal transport properties and contribute to the design of functional heterostructures [4,5,6]. In particular, it not only ensures the stability and epitaxial quality of two-dimensional materials, but also helps to improve the electronic, optical and topological properties of heterojunction [7,8]. Both graphene (Gr) and hexagonal boron nitride (h-BN) have six-membered ring, and the lattice constant between them is only 1.8% [9]. In addition, Gr has zero band gap, while h-BN has wide band gap [10,11]. It means that the heterojunction composed of Gr and h-BN may produce new properties [12]. In 2010, large-area atomic layers of h-BNC material were synthesized by L. Ci et al. [13]. Their findings indicate that band gap of the h-BNC is different from that of pure h-BN and Gr. In 2011, G. Seol et al. [14] found that the band gap of Gr/h-BN heterojunction can be regulated. The heterojunction can be transformed from semiconductor to semi metal or metal. Recently, Y. Qi et al. [15] found that the nucleation and growth of h-BN preferentially occur on the single crystal substrate, and the strong interfacial interaction is the key factor to induce this unique growth behavior. The results show that interface interaction can regulate the growth behavior of heterostructures. The previous studies have shown that boron nitride can open the band gap of graphene to a certain extent, and induce new thermal conductivity and electrical/magnetic properties [16,17]. The idea of using the in-plane Gr/h-BN under various conditions and for a variety of applications has engaged the minds of many researchers. T. H. Nguyen et al. [18] found that the electronic structure and properties of the in-plane Gr/h-BN heterostructure can be modulated by microscopic measurements. It provides a new method to study the catalytic properties of in-plane Gr/h-BN heterostructures and their defectivity. J. F. Zhang et al. [19] used first-principles to explore the atomic structures and electronic properties of interfaces in lateral Gr/BN heterostructures. Their findings indicate that Clar’s sextet rule plays a crucial role in the atomic structures and electronic properties of heterostructures. It provides a new method of tailoring the physical properties of heterostructures. In addition, the heterojunction can obtain excellent spin transport properties by various control methods. The introduction of defects has been proved to be an effective means. The existence of defects will lead to the localization of electron and phonon waves in two-dimensional materials. By introducing defects, the heterojunction shows good transport properties [20,21,22].

With the rapid development of nano-devices, the heat dissipation of circuits has become a bottleneck in the development of micro/nano-devices. Can Gr-hBN-PH inherit the excellent thermal conductivity of the former? What physical and chemical methods can be used to control its thermal conductivity? More importantly, how does the coupling of defect and strain affect the interfacial thermal transport of Gr-hBN-PH? 

It is very important to understand the effect of defects on the interfacial thermal transport of Gr-hBN-PH. In addition, we should consider how to use defects to reasonably control the thermal transport of two-dimensional materials.

In the present work, the defects (local stress generated) induces the formation of Gr-hBN-PH to form "unsteady structure". Then, the coupling effects of external field (heat flow direction, strain field and temperature field) and internal field (defect number, geometry shape and interfacial configuration) on the interfacial thermal transport of Gr-hBN-PH were studied.

## 2. Computational Model and Method

LAMMPS is a classical molecular dynamics code with a focus on materials modeling. It’s an acronym for Large-scale Atomic/Molecular Massively Parallel Simulator [23]. The molecular dynamic simulation in the present work has been described by using LAMMPS [23]. 

Vacancy formation energy of the Gr-hBN-PH was computed using Density functional theory (DFT), as implemented in the Vienna ab initio simulation package (VASP) [24]. The Perdew−Burke−Ernzerhof (PBE) functional [25] with the SVP basis set [26] were employed to optimize the geometrical structures of all studied models. In SVP, the inner shell atomic orbitals are described by a single basis function. A commonly used level of theory in the study of different nanostructures can be described by PBE [27]. In this calculation, the convergences in energy and force were set to 5 × 10^−6^ Eh and 3 × 10^−4^ Eh/Bohr.

A heat flow in the simulated model is performed by NEMD method. Heat flow is applied by exchanging kinetic energy in the "hot" region and the "cold" region [28]. The cold area is located at the left end of the system and the hot area is located in its right. In this way, the hot bath temperature increases and the cold bath temperature decreases. After a period of time, a stable temperature gradient is formed in the system. Exchanging kinetic energy during the process can be expressed by following formula:

(1) $$E=\sum_{N_{\mathrm{swap}}}\frac{1}{2}(m_{1}v_{\mathrm{hot}}^{2}{-m}_{2}v_{\mathrm{cold}}^{2})$$

(2) $$t_{total}\;{=N}_{swap}\times t_{swap}$$

(3) $$J(t)=\frac{1}{A}\sum_{N_{\mathrm{swap}}}\frac{1}{2}(m_{1}v_{\mathrm{hot}}^{2}{-m}_{2}v_{\mathrm{cold}}^{2})/t_{total}$$

**Figure 1:**
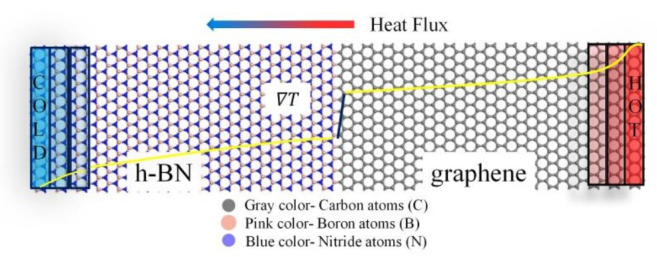
Set-up for the NEMD method. Heat flow is achieved by exchanging kinetic energy of slow moving particles in the “hot” region and fast-moving particles in the “cold” region. After a period of time, a stable temperature gradient is formed in the system.

In the above formula, $v_{\mathrm{hot}}^{}$ and $v_{\begin{array}{l} \mathrm{cold} \end{array}}^{}$ are the minimum particle velocity in the hot bath and the maximum particle velocity in the cold bath, respectively. The $m_{1}$ and $m_{2}$ are the mass of corresponding atoms in hot bath, and cold bath, respectively. $t_{total}$ is the total exchange time, and $t_{swap}$ is the exchange speed time interval. $N_{swap}$ is the number of particle exchange rates between cold bath and hot bath in each time step. J is the heat flow between the cold bath and the hot bath. Set-up for the NEMD method is shown in Figure 1.

The heat flux reaches a constant value when the steady state of system is achieved. In the simulations, the steady state region is established after 20 ns, corresponding to 4 × 10^6^ time steps. The system is first relaxed to an equilibrium state through three different steps. Firstly, the energy of system is minimized through the conjugate gradient method. Then, the simulated model is first equilibrated in the constant atom number, volume, and temperature ensemble (NVT ensemble) at 300 K for 5 ns. Next, the NVT ensemble is converted to NVE ensemble (constant volume and no thermostat), and the NVE ensemble is used to relax the 5 ns. Perform constant NVE is a system trajectory consistent with the microcanonical ensemble to update position and velocity for atoms in the each time step. The canonical (NVT) performs time integration on Nose-Hoover style non-Hamiltonian equations of motion. N is atom number; V is volume; E is energy; T is temperature.

**Figure 2:**
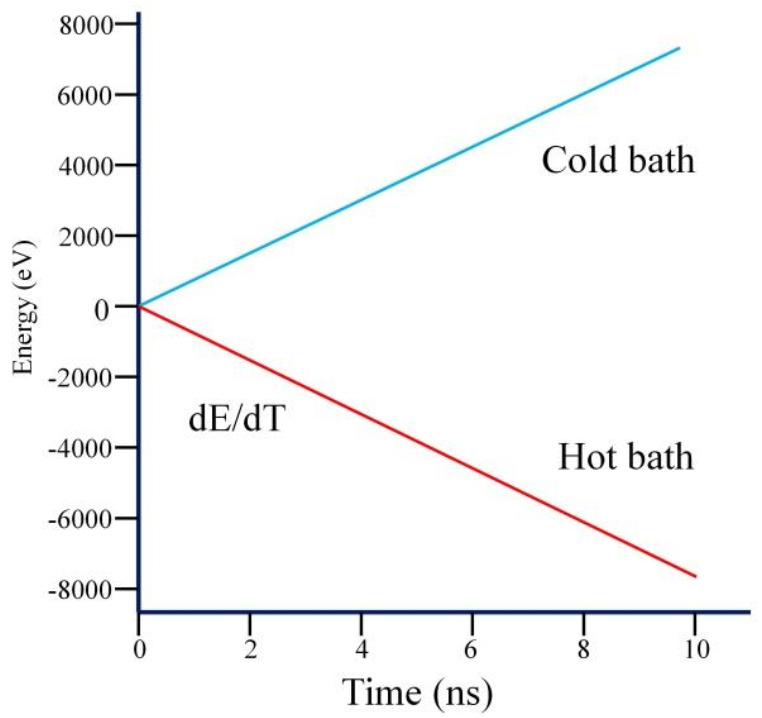
The accumulated energy of hot bath and cold bath with time.

Finally, the system temperature reaches about 300 K and fluctuates slowly around 300 K, indicating that the equilibrium stage is achieved. The accumulated energy of hot bath and cold bath with time was calculated, as shown in Figure 2. The energy curves are respected to the cross-section area. Furthermore, the accumulated energy of the thermostat (averaged over the heat source and sink) is a function of the time in steady state. The energy exchanged rate dE/dt is calculated as the slope of the linear fit. It is found that the sum of the increased/decreased energy is equal to 0, so the goal of conservation of total energy is achieved.

(4) $$T_{i}=\frac{2}{{3n}_{i}k_{B}}\sum_{j}\frac{p_{j}^{2}}{{2m}_{j}}$$

where $T_{i}$ is the temperature of the hot or cold bath, $n_{i}$ is the number of atoms in the region, and $k_{b}$ is the Boltzmann constant. $m_{j}$ and $p_{j}$ are the mass and momentum of atom j, respectively. The temperature gradient is calculated according to the average temperature of each region. When the heat flux and temperature gradient are constant, the thermal conductivity of the $L_{x}$ sample is obtained directly from Fourier law.

(5) $$\kappa (L_{x})=\frac{\langle J_{x}\rangle }{\nabla_{x}T}$$

$\nabla_{x}T$ is the arithmetic mean of the temperature gradient in the heat conduction directions.

(6) $$\nabla_{x}T=\frac{\left|\nabla_{x}T_{1}\right|+\left|\nabla_{x}T_{2}\right|}{2}$$

Due to the different electrical properties and atomic vibration modes of different materials, energy carriers (phonons or electrons) will scatter when passing through the boundary. The thermal resistance of the boundary will cause temperature change on both sides of the interface, thereby affecting heat dissipation, which is one of the main culprits of overheating problem. Therefore, how to reduce the interface thermal resistance has become a hot research topic. Due to the existence of Kapitza resistance, there is a significant temperature leap at the boundary. The temperature difference of the interface is obtained by linear fitting. The calculation formula of the ITC is:

(7) $$G=\frac{J_{x}}{\Delta T}$$

We assume that the thickness of graphene and h-BN is 3.3 Å (z direction).

## 3. Results and Discussion

### 3.1. Coupling Effects of Heat Flow Direction and Temperature

**Figure 3:**
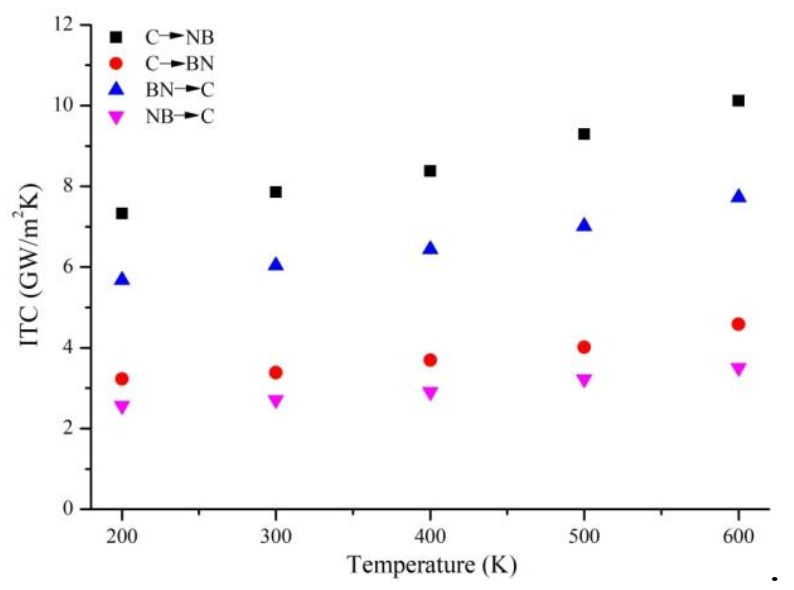
Temperature dependence of ITC based on the two kinds of heat flux direction.

For asymmetric Gr-hBN-PH, the ITC of the heterojunction is determined by heat flux direction. Therefore, we study the effects of heat flux direction and temperature on the ITC of Gr-hBN-PH. Figure 3 shows temperature dependence of ITC based on the two kinds of heat flux direction. The first is heat flow from Gr to h-BN, and another is from h-BN to Gr.

It is noted that C→NB shows the direction of heat flow from Gr to h–BN, and the interface is connected by C–N bond. C→BN shows the direction of heat flow from Gr to h–BN, and the interface is connected by C–B bond. BN→C shows the direction of heat flow from h–BN to Gr, and the interface is connected by C–N bond. NB→C shows the direction of heat flow from h–BN to Gr, and the interface is connected by C-B bond.

It is found from Figure 3 that the heat transfer efficiency from Gr to h–BN is higher than that from h–BN to Gr. With the increase of temperature, the difference becomes more significant. In addition, the degree of thermal rectification is raised with the increase of temperature discrepancy between cold source and heat source. It can be attributed the fact that the thermal conductivity of Gr is very susceptible to temperature, while the thermal conductivity of h–BN is not susceptible to temperature. When heat flow is transferred from Gr to h–BN, Gr works at a higher temperature. However, Gr is at a relatively low temperature under the opposite heat flow. Therefore, when heat flow is transferred from Gr to h-BN, the heat transfer efficiency of the heterostructure is higher.

It also found that the ITC of N–C bond is greater than that of B–C bond. Although C–B and C–N interactions are covalent in nature, the strength of C–N bond is greater than that of C–B bond, which indicates the advantage of C–N interface in phonon transport.

### 3.2. Coupling Effects of Interfacial Configuration and Temperature

**Figure 4:**
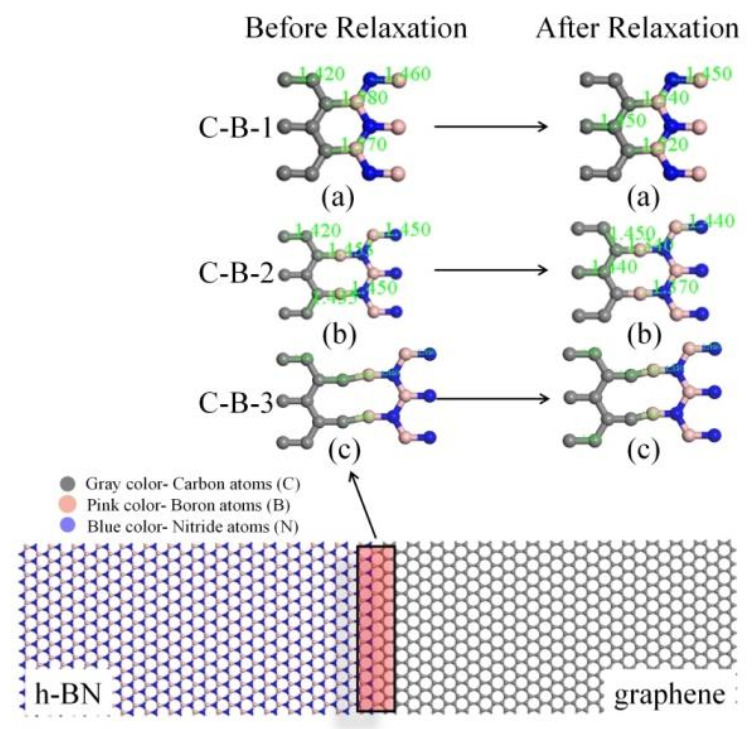
Gr-hBN-PH with different interfacial configuration.

In order to further study the coupling effect of interfacial configuration and temperature on the ITC of Gr-hBN-PH, we established the Gr-hBN-PH model with different interfacial configuration, as shown in Figure 4. We divided the interface into six categories: graphene/h–BN interface (C–B bond (interfacial configuration), C–B–1, C–B–2 and C–B–3) and (C–N–1, C–N–2 and C–N–3). 

**Figure 5:**
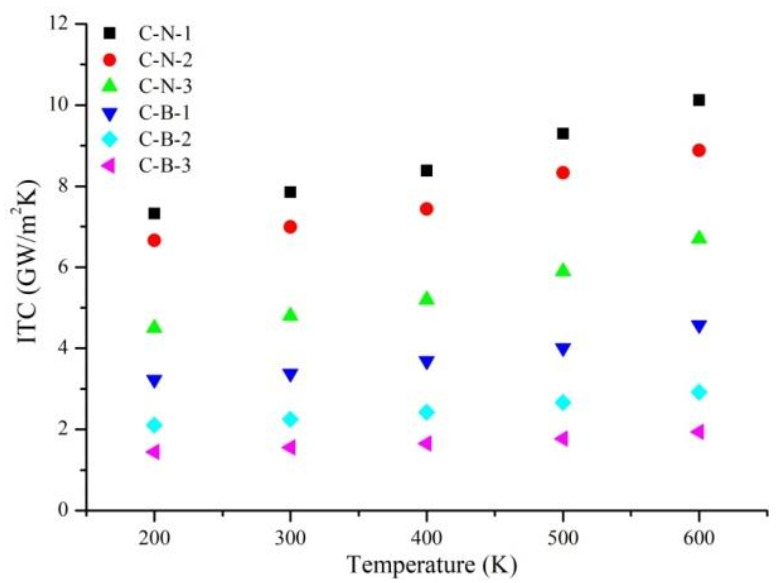
ITC of Gr-hBN-PH with different interfacial configuration.

Figure 5 show ITC of Gr-hBN-PH with different interfacial configuration. At 300 K, the ITC of sample C-N-1 is 7.8524 GW/m^2^K. The ITC of Gr-hBN-PH is much higher than that of other graphene-based planar heterostructures, such as graphene/silicon planar heterostructure (∼0.26 GW/m^2^K) and graphene/molybdenum disulfide (∼0.25 GW/m^2^K). It indicates that Gr-hBN-PH is an efficient heat transfer material. The ITC of Gr-hBN-PH increases with the increase of temperature, which is mainly determined by the inelastic scattering of phonons at the interface. The thermal relaxation time of phonons becomes smaller, and the inelastic scattering of phonons is enhanced. In addition, high temperature can arouse more high frequency phonons to participate in the ITC.

The interfacial configuration has a significant effect on the ITC of Gr-hBN-PH. N–C bond interfacial configuration has higher ITC than B–C interfacial configuration. When the temperature is 300 K, the ITC of C–N–1 is 7.8524 GW/m^2^K, which is greater than that of C–B–1 (3.3812 GW/m^2^K).

### 3.3. Coupling Effects of Defects Type and Heat Flow Direction

In the actual preparation process of Gr-hBN-PH, there are inevitably defects in the interface, especially vacancy defects. Does the existence of defects reduce the ITC of Gr-hBN-PH? What is the difference in the ITC of Gr-hBN-PH with different types of defects?

**Figure 6:**
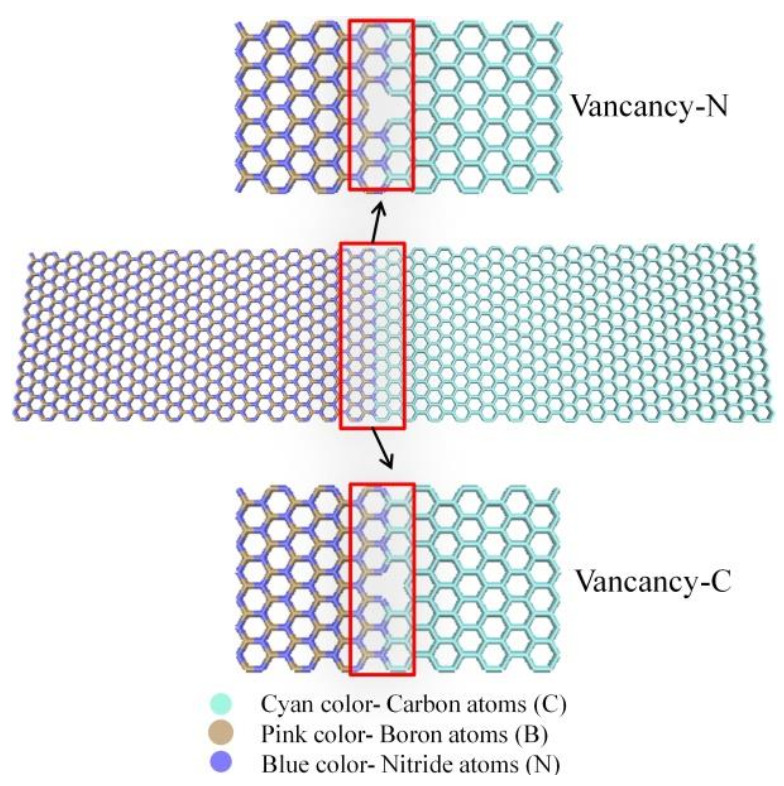
Two kinds of vacancy defects (Vancancy-C and Vancancy-N) are formed at the interface of Gr-hBN-PH.

**Figure 7:**
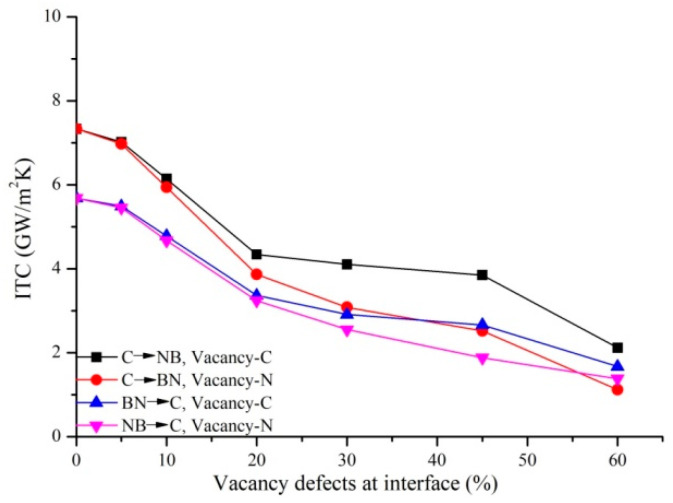
Coupling effect of two vacancy defects (nitrogen and carbon atoms) and heat flux direction on the ITC of Gr-hBN-PH.

Two kinds of vacancy defects (nitrogen atom and carbon atom) are created at the Gr-hBN-PH interface, as shown in Figure 6. The coupling effect of defect type and heat flow direction on the ITC of Gr-hBN-PH is studied, as shown in Figure 7. It is found that the ITC of Gr-hBN-PH decreases with the increase of defect concentration. When the defect concentration increases from 0% to 60% and the heat flux direction is from graphene to h-BN, the ITC of Vacancy-C decreases from 7.326 GW/m^2^K to 2.117 GW/m^2^K, which decreases by 71.10%. However, the ITC of Vacancy-N decreased from 7.326 GW/m^2^K to 1.121 GW/m^2^K, which decreased by 84.70%. The introduction of vacancy defects increases the phonon scattering in heterostructures. In addition, the existence of defects also affects the phonon transmission of the composite system, resulting in the decrease of the ITC of the interface. When the heat flux direction is the same, the ITC of Vacancy-C is higher than that of Vacancy-N. The lattice thermal conductivity is mainly based on the nonlinear interaction between phonons and the influence of defects. The strength of phonon interaction is closely related to chemical bond. The bond energies of C–C, N–B, C–N and C–B are 607, 389, 770, and 448 kcal/mol [29], respectively. The weak chemical bond in the system is usually characterized by low sound velocity and low bulk modulus. As the strong chemical bond is lost (increase the content of weak chemical bond), it will lead to the decrease of heat transfer efficiency. On the other hand, when the heat flux is transferred from graphene to h-BN, the heat flux on Vacancy-C is higher than that on Vacancy-N. The temperature gradient of nitrogen (caused by vacancy defect) is greater than that of carbon (caused by vacancy defect). Therefore, the ITC of planar heterojunction interface caused by two types of defects is different.

### 3.4. Coupling Effects of Defect Type and Strain

In the previous section, we studied the coupling effects of defects and heat flux direction on the interfacial thermal transport of Gr-hBN-PH. The stress changes the distance between atoms, which in turn changes the interaction between atoms. The lattice structure will also be changed from the "non-equilibrium structure to the final equilibrium", and then generate a new material form. What is the coupling effect of defect type and strain on the interfacial thermal transport of Gr-hBN-PH?

Two types of vacancy defects are created at the interface of the Gr-hBN-PH: Vacancy defects-C and Vacancy defects-N. Then, tensile strain, zero strain and compressive strain were applied along the length direction of Gr-hBN-PH. We define the strain of the Gr-hBN-PH as follows:

(8) $$\varepsilon =\frac{L-L_{0}}{L_{0}}$$

In the above formula, ε is the strain of the composite system; L is the length of the Gr-hBN-PH after deformation; and $L_{0}$ is the original length of the Gr-hBN-PH without deformation. It is noted that all heterostructures are subjected to a strain ranging from −0.15 to 0.15.

**Figure 8:**
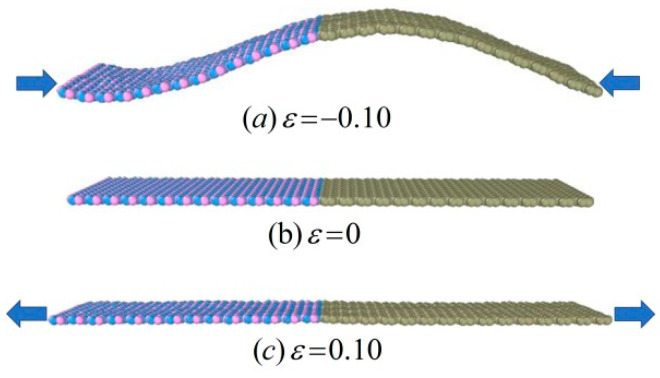
The morphology of the Gr-hBN-PH under different strain states along the length direction.

Figure 8 shows the morphology of the Gr-hBN-PH under different strain states along the length direction. Due to the low bending stiffness of the Gr-hBN-PH, the spatial configuration of system will change obviously under the action of compressive strain, showing a wave-like structure.

**Figure 9:**
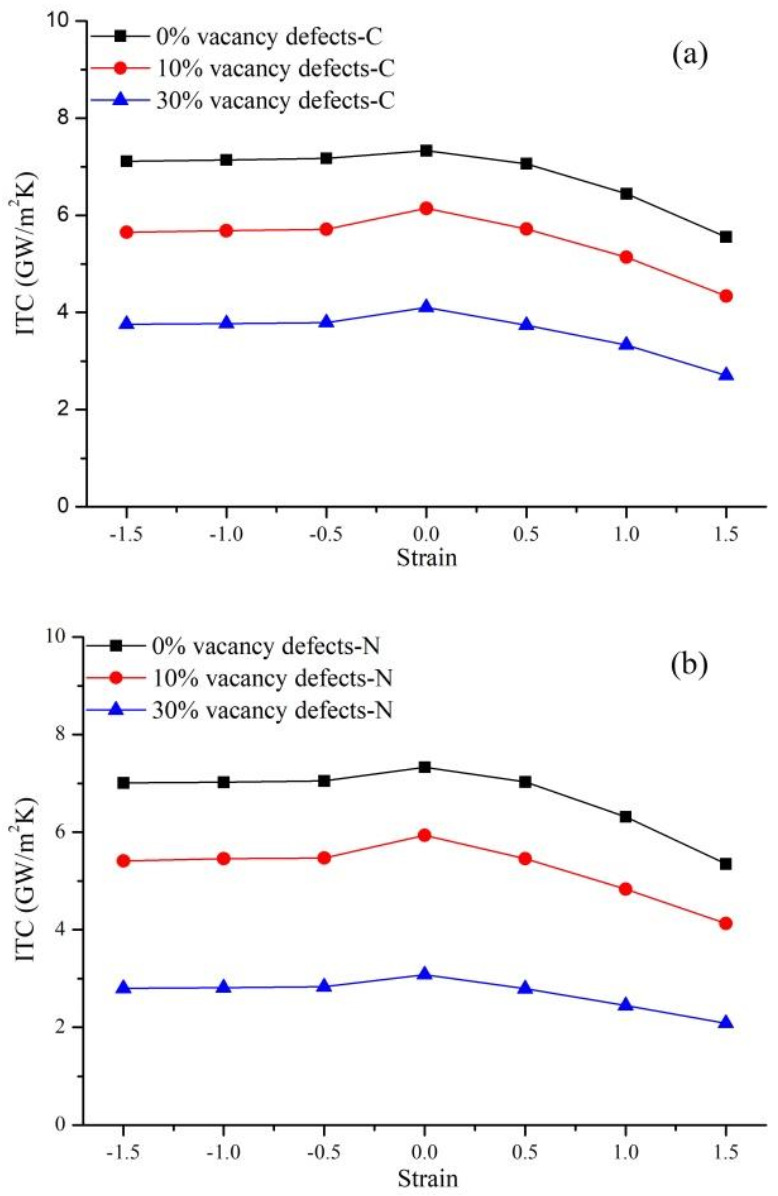
Coupling effects of defects and strain on the ITC of the Gr-hBN-PH. (**a**) Carbon vacancy defect (Vacancy defects-C); (**b**) nitrogen vacancy defect (Vacancy defects-N).

Figure 9 shows the coupling effects of defect type and strain on the ITC of the Gr-hBN-PH. It is shown that the ITC of the defective Gr-hBN-PH decreases slowly in the compressive strain range (0–0.5), while decreases rapidly in the tensile strain range (0.5–1.5). This is mainly because the bond length and bond angle of the defective Gr-hBN-PH change during the tensile deformation process, and the bond length increases with the increase of tensile strain. The nonharmonic interaction between atoms in the composite system is strengthened, leading to the softening of high frequency phonons. In addition, it also affects the phonon group velocity, reduces the phonon transmission efficiency, and ultimately reduces the interface thermal conductivity of the heterostructure.

The ITC of the defective Gr-hBN-PH changes relatively little when the compressive strain is applied in the length direction of system. For example, the ITC of defect-free Gr-hBN-PH only decreases by 5.68% when the compression deformation is 1.5. It can be attributed to the fact that due to the special two-dimensional structure of graphene and h-BN, the composite system releases part of the stress by forming wave arches during compression deformation. In addition, the bond length and bond angle of the defective Gr-hBN-PH will not deform significantly during compression deformation, which is also the reason why the interface ITC does not change much.

### 3.5. Coupling Effects of Defect Geometry and Strain

**Figure 10:**
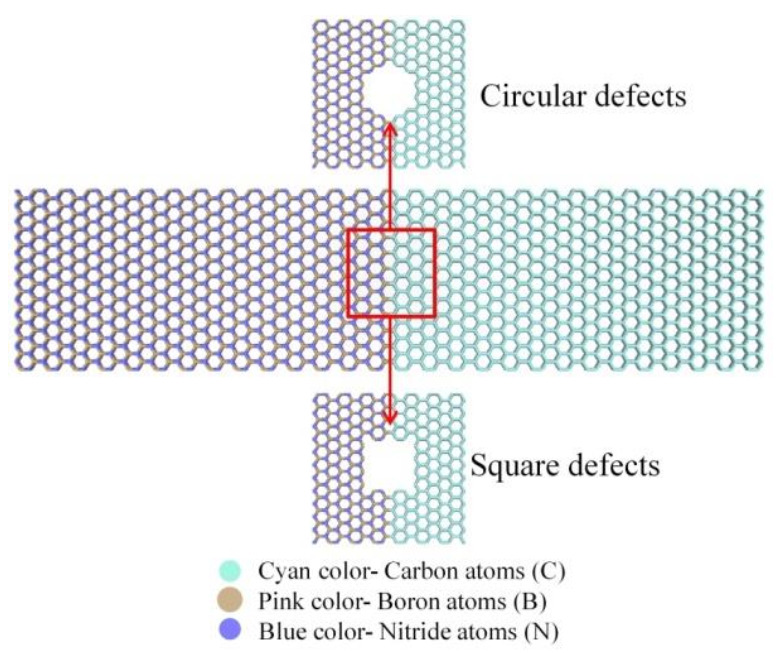
The interface of the Gr-hBN-PH with two geometric types of vacancy defects, including circular defect and square defect.

Two geometric types of vacancy defects are generated at the interface of the Gr-hBN-PH: Circular defect and square defect, as shown in Figure 10. Then, tensile strain, zero strain and compressive strain were applied along the length direction of the Gr-hBN-PH.

**Figure 11:**
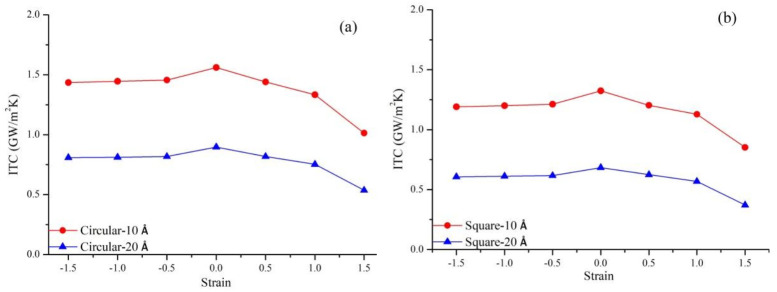
Coupling effects of geometric defects and strain on the ITC of the Gr-hBN-PH. (**a**) circular defect; (**b**) square defect.

Figure 11 shows the coupling effect of geometric defects and strain on the ITC of the Gr-hBN-PH. The existence of geometric defects decreases ITC of the Gr-hBN-PH. Besides, compressive strain has little effect on the ITC of the Gr-hBN-PH with geometric defects, while tensile strain has a greater effect on the ITC of the Gr-hBN-PH with geometric defects.

The effect of circular defects on the ITC of the Gr-hBN-PH is less than that of square defects under the coupling of geometric defects and strain. It can be attributed to the fact that system with the square defect is easy to cause stress concentration, which leads to the change of bond length and bond angle. These changes will lead to the decrease of ITC. Another reason is that the loss of atom and bond energy (caused by square defect) is greater than that of circular defect under the same conditions. The more six-membered rings are destroyed, and thus more covalent bonds are eliminated, the greater reduction of the ITC of heterojunction will be.

### 3.6. Phonon Density of States Analysis

In order to further explain the thermal transport of Gr-hBN-PH. The density of phonon states (PDOS) at the interface is obtained by Fourier transform of the velocities of carbon (C), boron (B) and nitrogen (N) atoms in the region,

(9) $$P(\omega )=\frac{1}{\sqrt{2\pi }}\int_{-\infty }^{\infty }VACF(t)dt$$

where ω is the frequency of the vibration wave, and VACF(t) is the autocorrelation function.

The autocorrelation function VACF(t) can be expressed as follows:

(10) $$VACF(t)=\frac{1}{N}\sum_{i=1}^{N}\langle v_{i}(0)v_{i}(t)\rangle$$

$V_{i}(t)$ is the velocity vector of particle i at time t, and N is the atomic number of the system. The sampling rate is performed every 5 fs. Three kinds of phonon spectra (LA,TA and ZA) are obtained by Fourier transform of carbon, boron and nitrogen atoms. 

**Figure 12:**
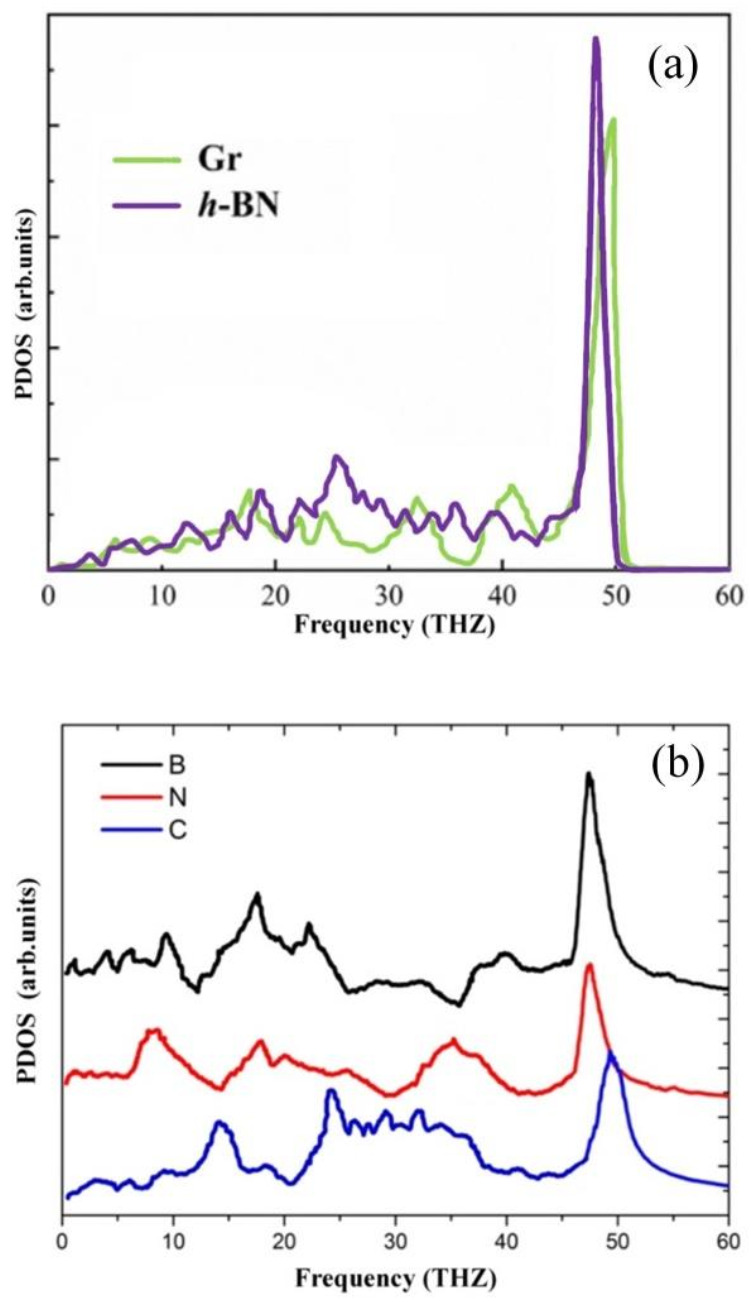
PDOS of the different systems (**a**) graphene and h–BN domain, (**b**) Gr-hBN-PH.

It is found from Figure 12a that the PDOS overlaps of graphene and h-BN are distributed in the overall frequency range, especially at the low frequency (<20 THz). From the point of view of lattice dynamics, the phonon transport at the interface between the two materials strongly depends on the overlap of PDOS, i.e., high ITC, indicates that phonons are easier to pass through the interface. Figure 12b shows the PDOS of the Gr-hBN-PH. It is noted that C atom belongs to graphene, B and N atoms belong to hexagonal boron nitride. For the Gr-hBN-PH, the PDOS of N atom overlaps that of C atom more than that of boron atom. Therefore, we can conclude that the C–N bonding at interface has more effective interfacial thermal transport than C–B bonding at interface.

Therefore, if we want to control the interfacial thermal transport of Gr-hBN-PH, it is better to consider these interfacial configurations in the interface of Gr-hBN-PH in order to explore new physics and applications.

### 3.7. Defect Energy Analysis

The properties of two-dimensional materials are closely related to materials structure, atoms interaction and external environment. Therefore, we analyze the characteristics of Gr-hBN-PH from the perspective of energy. The vacancy formation energy is calculated as follows:

(11) $$E_{v}=E_{defect}-(N-X/N)E_{prefect}-\eta_{x}\mu_{C}-\eta_{y}\mu_{B}\;-\eta_{z}\mu_{N}$$

where, N is the total number of particles, X is the defect vacancy; $E_{prefect}$ is the total energy of the optimized unit cell, $E_{defect}$ is the total energy of the optimized unit cell with vacancy, $\eta_{i}$ is the number of boron, nitrogen or carbon atoms removed to produce defects and $\mu_{i}$ is the chemical potential of the i atom.

**Figure 13:**
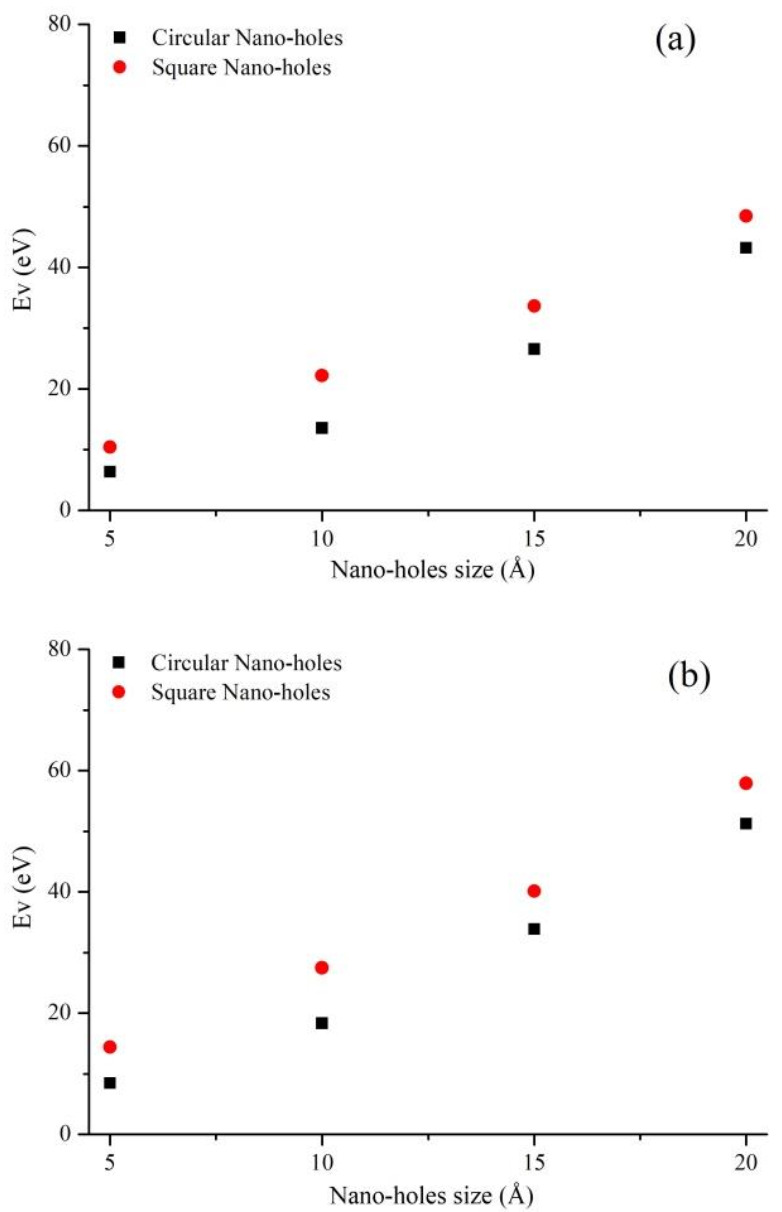

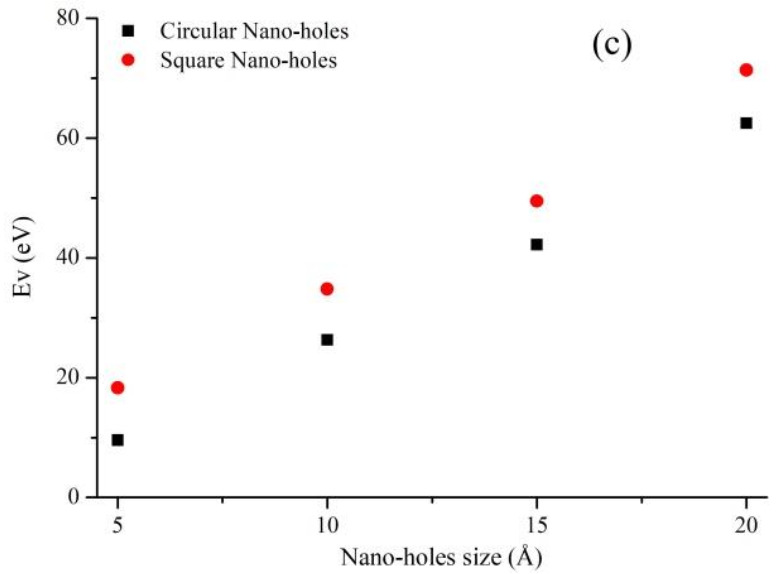
Vacancy formation energy of Gr-hBN-PH. The defects are created in (**a**) graphene region, (**b**) interface region, and (**c**) boron nitride region, respectively.

It can be seen from Figure 13 that the vacancy formation energy of circular defect is less than that of square defect. When the defect size is 5 Å, the vacancy formation energy of circular defect (Interface) is 8.445 eV, while that of square defect (Interface) is 14.353 eV. It can be attributed the fact that the atomic loss of square defect is greater than that of circular defect. The energy required for defects in graphene region is greater than that in h-BN region. Therefore, defects are more likely to form in the h-BN region. The physical mechanism between structure and properties in the low dimension is analyzed from the perspective of defect energy. 

## 4. Conclusions

In this work, the defects (local stress generated) induces the formation of Gr-hBN-PH to form "unsteady structure". Then, the coupling effects of external field (heat flow direction, strain field and temperature field) and internal field (defect number, geometry shape and interfacial configuration) on the interfacial thermal transport of Gr-hBN-PH were studied.

The ITC of the Gr-hBN-PH increases with the increase of temperature. The interface bonding morphology has a significant effect on the ITC of the Gr-hBN-PH. The Gr-hBN-PH with N–C bond interface has higher ITC than B–C interface. The PDOS of N atom overlaps that of C atom more than that of boron atom. Therefore, we can conclude that the C–N bonding at interface has more effective interfacial thermal transport than C–B bonding at interface.

Under the action of compressive strain, the spatial configuration will change obviously, and the structure shape will be similar to wave. The bond length and bond angle of the Gr-hBN-PH do not show significant deformation. The thermal transport at the interface is less affected by compression deformation. However, under tensile deformation, the morphology of the heterostructure is smooth, the surface wrinkles are almost disappeared, and the bond length and bond angle of the Gr-hBN-PH will change. The enhancement of non-harmonic interaction between atoms in the composite system leads to the softening of high-frequency phonons, which affects the phonon group velocity, thus reducing the phonon transmission efficiency and reducing the thermal transport at the interface of heterostructures.

## Data Availability

Data sharing not applicable.
